# Halobacterial nano vesicles displaying murine bactericidal permeability-increasing protein rescue mice from lethal endotoxic shock

**DOI:** 10.1038/srep33679

**Published:** 2016-09-20

**Authors:** Arjun Balakrishnan, Priya DasSarma, Oindrilla Bhattacharjee, Jong Myoung Kim, Shiladitya DasSarma, Dipshikha Chakravortty

**Affiliations:** 1 Department of Microbiology and Cell Biology, Indian Institute of Science, Bangalore, India; 2 Institute of Marine and Environmental Technology and Department of Microbiology and Immunology, University of Maryland, Baltimore, MD, USA; 3 Center for Biosystem Science and Engineering, Indian Institute of Science, Bangalore, India

## Abstract

Bactericidal/permeability-increasing protein (BPI) had been shown to possess anti-inflammatory and endotoxin neutralizing activity by interacting with LPS of Gram-negative bacteria. The current study examines the feasibility of using murine BPI (mBPI) expressed on halophilic Archaeal gas vesicle nanoparticles (GVNPs) for the treatment of endotoxemia in high-risk patients, using a murine model of D-galactosamine-induced endotoxic shock. *Halobacterium* sp. NRC-1was used to express the N-terminal 199 amino acid residues of mBPI fused to the GVNP GvpC protein, and bound to the surface of the haloarchaeal GVNPs. Our results indicate that delivery of mBPIN-GVNPs increase the survival rate of mice challenged with lethal concentrations of lipopolysaccharide (LPS) and D-galactosamine. Additionally, the mBPIN-GVNP-treated mice displayed reduced symptoms of inflammation, including inflammatory anemia, recruitment of neutrophils, liver apoptosis as well as increased pro-inflammatory serum cytokine levels.

Sepsis is a clinical condition arising from complex inflammatory responses to infection by the host. According to CDC’s National Center for Health Statistics (NCHS), the number of patients admitted to hospitals due to sepsis has increased from 621,000 in the year 2000 to 1,141,000 in 2008^1^. Due to the high mortality rates (28 to 50%) associated with sepsis, it remains one of the major concerns among high-risk patients^2^. The causative agent for sepsis can be Gram-negative bacteria, Gram-positive bacteria, or fungi. Recent reports on septic conditions found in ICU patients suggested a higher prevalence of Gram-negative bacterial infection than Gram-positive bacteria and fungi^3^.

Gram-negative bacteria-induced inflammation is mediated by the interaction of lipopolysaccharides (LPS) with Toll-like receptor 4 (TLR4)^4^. The outcome of such interactions is a rapid release of pro-inflammatory cytokines which can lead to recruitment of neutrophils to the site of infection, aggravating the condition unless LPS molecules are cleared from circulation. Under normal conditions, LPS from the gut lumen infiltrate the blood and are cleared by Kupffer cells present in the liver^5^. Conditions that lead to increased activity of Kupffer cells can provoke inflammation as observed in alcoholic hepatitis^6^. Therapeutic strategies have been formulated to target septic shock, including targeting TNFα using TNFα-specific antibodies or different stages of complement activation (Edaravone) and blood clotting (Thromboxane Inhibitors)^7^.

One of the well-studied endotoxin neutralizing molecules are BPI (bactericidal/permeability increasing protein). BPI is a 55 kDa glycoprotein primarily expressed in azurophilic granules of neutrophils in humans^8^. Expression of BPI is conserved throughout the course of evolution from invertebrates to vertebrates^9^. The anti-inflammatory properties of BPI are attributed to the interaction of the amino-terminal portion of the protein with the lipid A moiety of LPS, preventing the interaction of LPS and TLR4^10^. Recombinant human BPI (rBPI 21) containing the 199 N-terminal amino acids of BPI has been shown to have anti-inflammatory properties^11^, with potent therapeutic potential in phase III clinical trials. Despite having therapeutic potential, the high cost, and short half-life may make rBPI21prohibitive for wide-spread use.

In this study, we have attempted to overcome these limitations by displaying the N- terminal 199 amino acids of murine BPI (mBPI) on gas vesicle nanoparticles (GVNPs) from halophilic Archaea. The GVNPs are produced in an archaeon lacking LPS and are easily purified by flotation and have a long half-life. Additionally, a previous study from our group has shown that GVNPs by themselves do not cause inflammation in mice^12^,^13^. These properties together, make GVNPs an ideal candidate for displaying potential therapeutic agents, and in particular, BPI for the use against endotoxic shock. Our results indicate that mBPI displayed on haloarchaeal GVNPs (mBPIN-GVNPs) have significant protection in a mouse model of endotoxic shock. mBPIN-GVNPs also protect mice from LPS-induced inflammatory anemia and apoptosis of hepatocytes in D-galactosamine-sensitized mice.

## Results

### BPI gene synthesis and expression in *Halobacterium*

**Figure 1:**
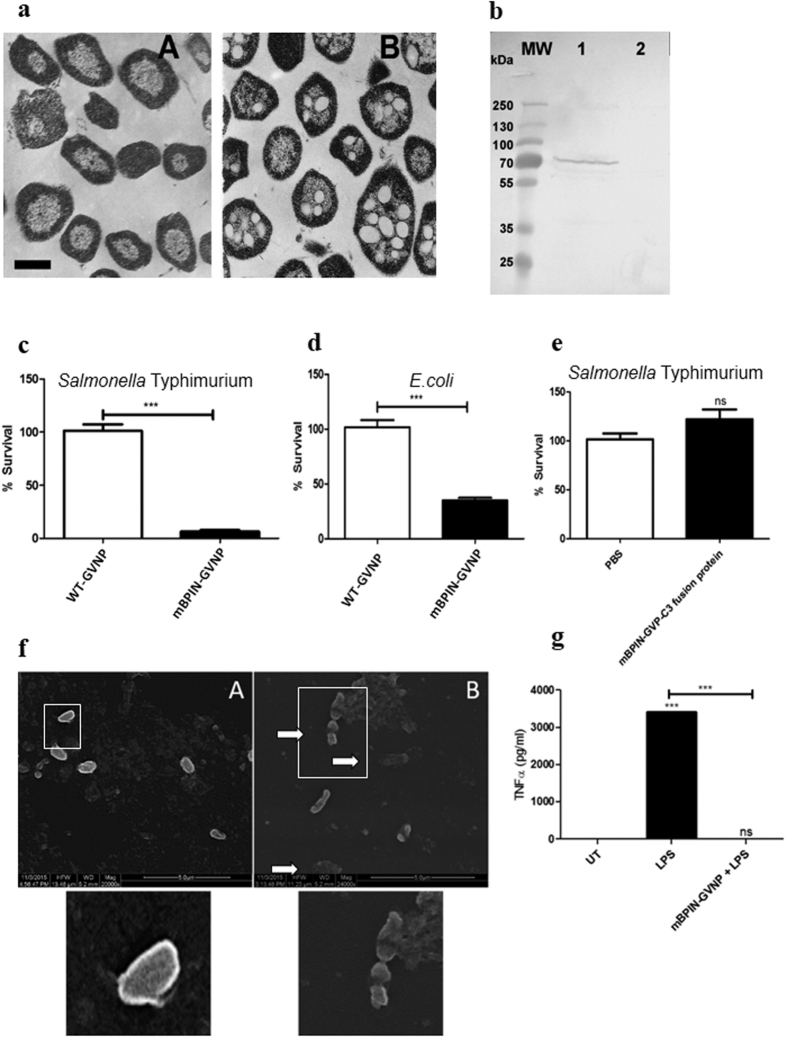
Expression of GvpC-mBPI N fusion protein in haloarchaeal gas vesicle nanoparticles and its antibacterial and anti-inflammatory activity. (**a**) Thin-sections of *Halobacterium* sp. observed by transmission electron microscopy Panel A. Strain SD109 with a deletion of the *gvp* gene cluster and lacking GVNPs. Panel B. Strain SD109 transformed with a plasmid containing the entire *gvp* gene cluster producing buoyant GNPVs. Bar in Panel A indicates 0.5 um length (for both panels) (**b**) *Halobacterium* sp. NRC-1 (pDRK-C3-mBPI) BPI-GVNPs were produced and purified by floatation of the GVNP particles using the accelerated centrifugation and expression of GvpC-mBPINfusion protein was confirmed by Western blotting using a 1:1000 dilution of anti-His-tag primary antibody. Lane 1: mBPIN-GVNPs, Lane 2: WT-GVNPs. 10^6^ *Salmonella* Typhimurium 14028 (**c**) and *E. coli* (**d**) were incubated with WT-GVNPs (White Bars) or mBPIN-GVNPs (Black Bars) for 2 hours at 37 °C. Cells were plated on LB agar and incubated at 37 °C overnight. Percent survival values are plotted and are means of triplicate assays. (n = 3 experiments) (**e**) 10^6^ *Salmonella* Typhimurium 14028 were incubated with PBS (White Bars) or mBPIN-GVP-C3 fusion protein (Black Bars) for 2 hours at 37 °C. Cells were plated on LB agar and incubated at 37 °C overnight. Percent survival values are plotted and are means of triplicate assays. (n = 3 experiments) (**f**) 10^6^ *Salmonella* Typhimurium 14028 were incubated with WT-GVNPs (Panel A) or mBPIN-GVNPs (Panel B) for 2 hours at 37 °C. Cells were fixed and observed by scanning electron microscopy. White arrow indicates membrane damage and leakage of cytosolic contents in bacteria incubated with mBPIN-GVNPs. Inserts were magnified to show cell morphology of WT-GVNP treated and mBPIN-GVNP treated bacteria. (**g**) PBMCs were treated with LPS (10 ng) or with LPS (10 ng) preincubated with mBPIN-GVNP. 24 h post treatment, supernatant was collected and TNFα levels were measured by ELISA. TNFαlevels were compared with un-treated control. (n = 3 experiments). Data was analyzed by students T test. Key: ***p < 0.001, **P < 0.01, *p < 0.05, ns = not significant.

We cloned a synthetic, codon-optimized N-terminal 199 AA of the mouse BPI (mBPIN) protein fragment into the *Halobacterium* pDRK-C3 overexpression vector, to construct plasmid pDRK-C3-mBPI. This plasmid was designed to produce a polyhistidine-tagged-GvpC3-Bpi fusion-protein binding to the surface of GVNPs. The constructed plasmid was transformed into *Halobacterium* sp. SD109, for the production of “free” fusion-protein, or *Halobacterium* NRC-1 hosts for display on the GVNP surface. GVNPs observed by TEM are visible as lemon-shaped organelles produced in cells (Fig. 1a).

Expression of mBPIN-GVP-C3 fusion protein was confirmed by Western blotting using an anti-His-tag antibody (Cell Signaling Technology, Danvers, MA) and by using alkaline phosphatase-conjugated goat anti-rabbit secondary antibody (Sigma-Aldrich, St. Louis, MO) (Fig. 1b). The fusion protein has shown migration at 70 kDa instead of 50 kDa. The observed anomaly of the size of the fusion protein is the result of its low pI, which is known to retard migration in SDS-PAGE^14^.

### HaloarchaealGVNPs displaying mBPIN show anti-bacterial and anti-inflammatory activity

BPI is known to possess specific anti-bacterial activity against Gram-negative bacteria^15^. This specific activity is attributed to a high binding affinity of the BPI for the Lipid A moiety of Gram-negative bacterial outer membranes^15^,^16^. To examine the anti-bacterial activity of mBPIN displayed on the surface of *Halobacterium* GVNPs, Gram-negative bacteria (*Salmonella* Typhimurium and *E. coli)* were incubated with 100 μg/ml of wild-type (WT)-GVNPs or mBPIN-GVNPs (which contain approximately 3 μg/ml of mBPIN-GVP-C3 fusion protein protein) and the anti-bacterial activity was assayed. mBPIN-GVNPs significantly inhibited the growth of *Salmonella* Typhimurium and *E. coli* (Fig. 1c,d). Bacterial numbers were reduced by 50 to 75% in the presence of mBPIN-GVNP compared to that of WT-GVNP showing a functionally active protein. Interestingly, under similar conditions, purified mBPIN-GVP-C3 fusion protein even at a 2x-higher concentration (6 μg/ml of mBPIN-GVP-C3 fusion protein) did not inhibit the growth of *Salmonella* Typhimurium (Fig. 1e). These results suggest that mBPIN-GVP-C3 fusion protein expressed on the surface of halo bacterial are functionally more active that the ‘free protein’. To see bacterial damage associated with BPI activity, 10^6^ *Salmonella* Typhimurium were incubated with 100 μg/ml of wild-type (WT)-GVNPs or mBPIN-GVNPs for 2 hours. Cells were fixed and cell damage was analyzed using scanning electron microscopy. Scanning electron microscopy analysis showed a significant amount of cell lysis and membrane perturbations upon incubation with mBPIN-GVNPs (Fig. 1f). In order to evaluate the *in vitro* anti-inflammatory property of mBPIN-GVNPs, PBMCs were treated with LPS or a combination of mBPIN-GVNPs and LPS. 24 h post treatment, the cell supernatant was collected and TNFα levels were quantified by ELISA (Fig. 1g). TNFα levels were significantly higher in LPS treated set compared to untreated control. Interestingly, mBPIN-GVNPs significantly reduced LPS induced TNFα secretion compared to untreated control. These experiments prove that mBPIN-GVNPs are functionally active.

### mBPIN-GVNPs protect mice from lethal endotoxic shock

**Figure 2:**
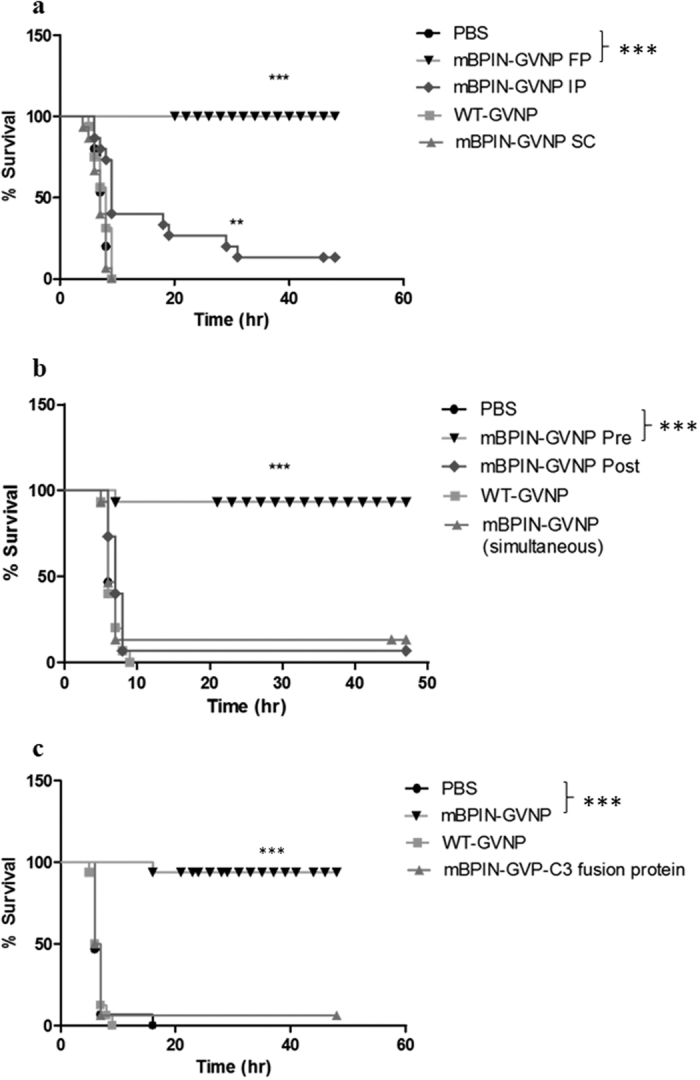
mBPIN-GVNPs protect mice from lethal endotoxic shock. Cohorts of 15 mice were injected with PBS, WT-GVNPs (100 μl of 1 mg/ml), or mBPIN-GVNPs (100 μl of 1 mg/ml) and endotoxic shock was induced using a combination of LPS and D-galactosamine following (**a**) Different routes of immunization: Subcutaneously (SC), Intraperitoneally (IP), or via Foot Pad (FP); (**b**) Foot pad injection pre (before 1 h) or post (after 1 h) LPS and D-galactosamine -treatment; (**c**) Foot pad injection of “free” mBPIN-GVP-C3 fusion protein (100 μl of 60 μg/ml) or mBPIN-GVNPs (100 μl, containing approximately a total of 30 μg/ml mBPIN-GVP-C3 fusion protein) 1 h before LPS and D- galactosamine treatment. Survival of mice was followed for 5 days. Statistical analysis were made in comparison with the survival rates of PBS treated control and the survival curves were compared using Log-rank-test. Key: ***p < 0.001, **p < 0.005.

To evaluate the anti-inflammatory properties of mBPIN-GVNPs (*in-vivo*), mice were injected with mBPIN-GVNPs through subcutaneous (SC), footpad (FP) or intraperitoneal (IP) routes. One hour post-injection, mice were intraperitoneally challenged with a lethal dose of LPS and D-galactosamine. All mice that were injected with WT-GVNPs or mBPIN-GVNPs administered subcutaneously died within seven hours after LPS treatment. However, mice that received mBPIN-GVNPs via the footpad route showed 100% survival (Fig. 2a). When mBPIN-GVNPs were injected intraperitoneally the life span of mice was extended by twenty-four hours. However, most of the mice (except one) had died by thirty hours, post LPS treatment (Fig. 2a). This difference in survival time due to different routes of administration may be due to the difference in absorption rates into the bloodstream of the particles^17^.

Footpad injection of mBPIN-GVNPs simultaneously with LPS does not protect mice from lethal endotoxic shock (Fig. 2b). This finding proves that mBPIN-GVNPs may need to be present in the circulatory system by pretreatment for complete clearance of LPS. The therapeutic effect of mBPIN may be dose-dependent, with more and higher stability forms being needed for better effectiveness. Injection of purified mBPIN-GVP-C3 fusion protein, even at a 2x-higher concentration 1h prior to LPS treatment did not show any protection (Fig. 2c). These findings prove that mBPIN protein displayed on the surface of GVNPs is functionally active and can protect mice from lethal endotoxic shock compared to mBPIN-GVP-C3 fusion-protein.

### mBPIN-GVNPs inhibit inflammatory-response mediated by LPS

**Figure 3:**
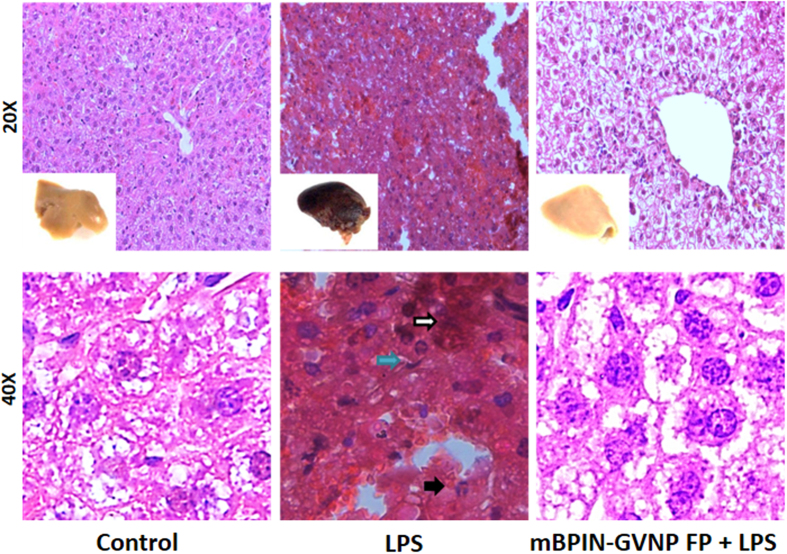
mBPIN-GVNPs protect mice from severe hepatic injury. (**a**,**d**) Untreated control; (**b**,**e**) LPS and D-galactosamine treated and (**c**,**f**) Mice were injected with mBPIN-GVNPs (100 μl of 1 mg/ml) via the foot pad 1 h prior to treatment followed by a combination of LPS and D-galactosamine. 6 hours post LPS and D-galactosamine-treatment, mice were euthanized and liver sections were stained using hematoxylene and eosine. Representative images are shown for each group (n = 5 mice/group) Top row: 20x magnification of liver thin-section, bottom row: 40x magnification of liver thin-section. Inserts: right liver lobe (post) treatment. Neutrophil infiltration (black arrow), Kupffer cells (blue arrow) and hemorrhage (white arrow).

**Figure 4:**
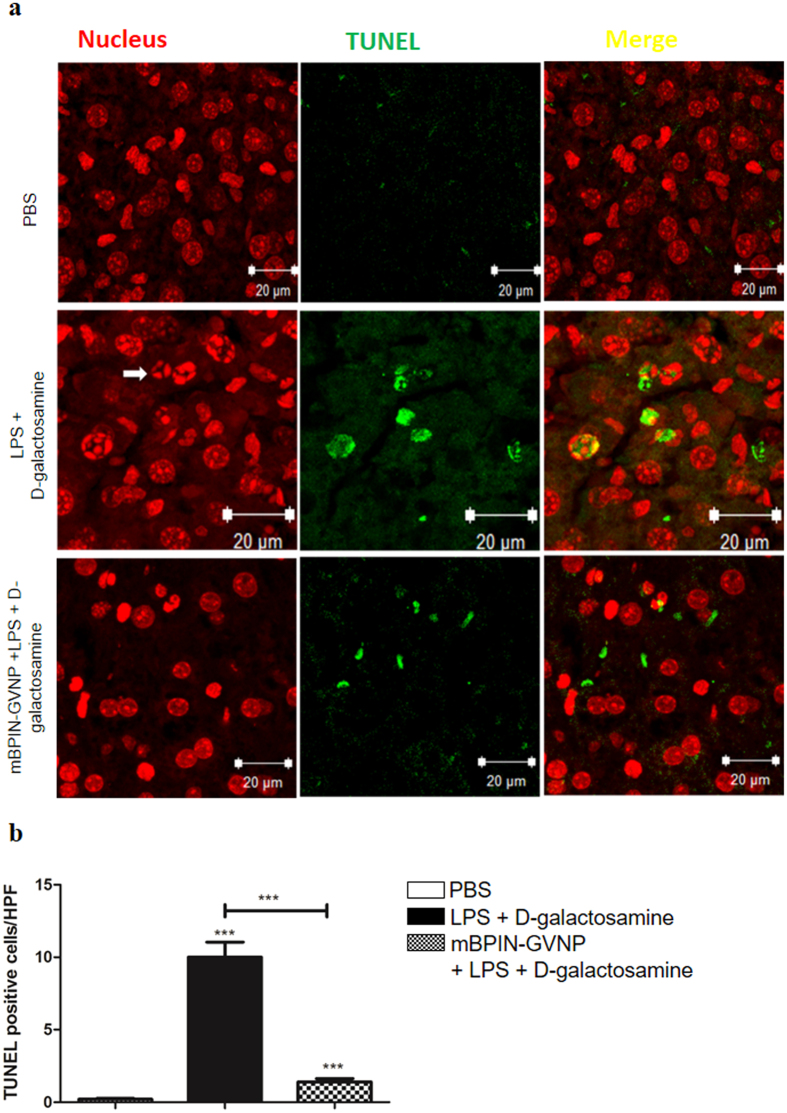
mBPIN-GVNPs diminish LPS and D-galactosamine-induced apoptosis in liver. (**a**) Mice were either untreated (top row), LPS and D-galactosamine-treated (middle row) or pretreated via injection through foot pad with mBPIN-GVNPs (100 μl of 1 mg/ml) followed an hour later, with LPS and D-galactosamine-treated (bottom row) followed by euthanasia 6 hours later. Liver sections were stained for identifying live cells, via propidium iodide (left column), Dead End TUNEL assay for identifying DNA fragmentation (center column) and images overlapped (right column). White arrows indicate neutrophil infiltration. Representative images are shown (n = 5 mice/group) (**b**) Quantitation of TUNEL positive cells. PBS treated (white bars), LPS and D-galactosamine (black bars), mBPIN-GVNPs via foot pad injection 1 hour before LPS treatment (checked bars). Key: ***p < 0.001.

LPS-mediated endotoxic shock is known to cause a number of effects in the murine host. We used D-galactosamine treatment, which primarily affects the liver in order to predispose the liver to deleterious effects of endotoxins^18^. The primary analysis of liver samples from LPS-treated mice revealed severe blood clotting and hypoxemia characterized by the darkening of liver compared to those of untreated mice. Histopathological analysis, including hematoxylin and eosin staining of liver section from LPS and D-galactosamine-treated mice also showed signs of inflammation along with recruitment of neutrophils, enlarged Kupffer cells and immense hemorrhaging in the liver samples (Fig. 3). mBPIN-GVNP administration prior to LPS treatment, notably repressed the deleterious effects of LPS on the liver. Liver samples from mBPIN-GVNP treated mice were normal in their texture (reddish brown in color) and did not show any inflammation. LPS along with D-galactosamine treatment is known to induce apoptosis in liver^19^. Analysis of liver sections for apoptosis showed a significant increase of apoptotic cells in LPS and D-galactosamine-treated mice as determined by the TUNEL assay (Fig. 4a, middle row). Treatment of mice with mBPIN-GVNPs significantly reduced LPS-induced apoptosis in the liver (Fig. 4a, bottom row). Quantitation of TUNEL positive cells showed a 3-fold difference in TUNEL positive cells between LPS treated set and mBPIN- treated set (Fig. 4b). These results indicate that BPI-GVNPs can inhibit deleterious effects of endotoxins in the liver.

**Figure 5:**
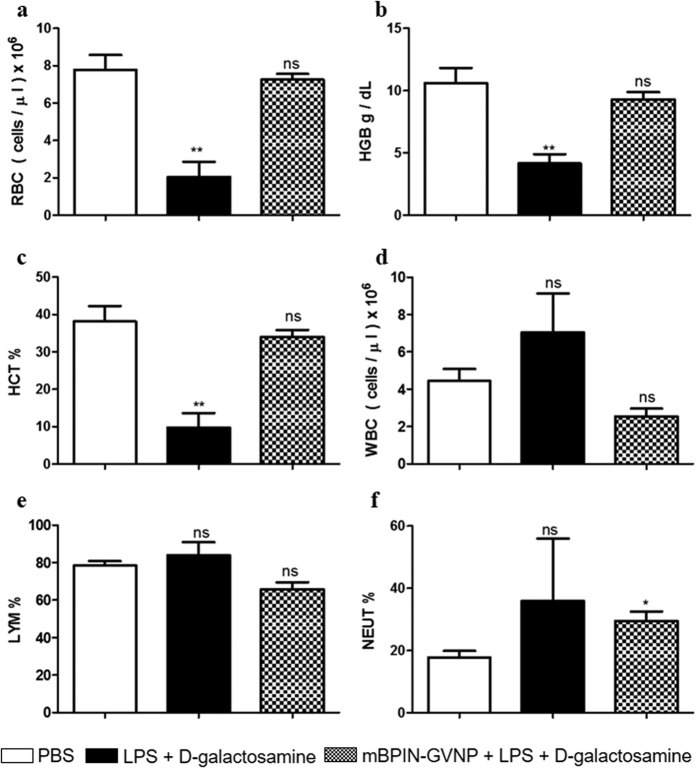
mBPIN-GVNPs reduce inflammatory anemia in mice. Cohorts of 5 mice were injected with PBS treated (white bars) or with injected LPS and D-galactosamine (black bars) or with mBPIN-GVNPs (100 μl of 1 mg/ml) via foot pad injection 1 h before LPS and D-galactosamine (8 mg/mouse)-treatment (checkered bars). 6 hours later, blood was collected and analyzed using an automated hematology analyzer. (**a**) Redblood cell count; (**b**) Hemoglobin; (**c**) Packed cell volume; (**d**) White blood cell count; (**e**) % Lymphocytes (**f**) % Neutrophils. Statistical analysis were made in comparison with the PBS treated control. Key: **p < 0.01, *p < 0.05, ns = not significant.

Endotoxins are known to induce inflammatory cytokine production, which leads to various pathophysiological conditions including anemia in patients^20^. Analysis of whole blood parameters revealed a 4-fold reduction in red blood cell counts (Fig. 5a) and a 2- fold reduction in blood hemoglobin levels (Fig. 5b) in the case of LPS treated mice compared to PBs treated control. Hematocrit or packed cell volume (Fig. 5c) was also consistently reduced by 4-fold in LPS treated mice compared to untreated control. Pre-administration of mBPIN-GVNPs and subsequent challenge with LPS-D-galactosamine treatment resulted in both RBC and hemoglobin levels equal to, or on par with untreated mice showing that mBPIN-GVNPs can reduce or even prevent endotoxin-induced anemia in mice. Other blood parameters including WBC count (Fig. 5d), and percentage of lymphocyte and neutrophil (Fig. 5e,f) were not significantly altered upon endotoxic shock, even though we see a tendency towards increased neutrophil count in LPS and mBPIN-GVNP treated group.

**Figure 6:**
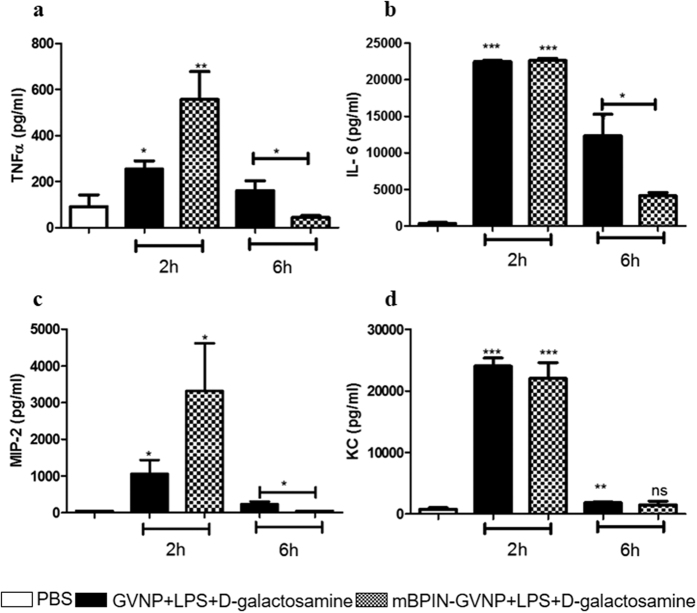
mBPIN-GVNPs decreased inflammatory cytokine levels in the blood during later stages of inflammation. Cohorts of 5 mice were either injected with PBS (white bars) or first pre-treated with WT-GVNPs (100 μl of 1 mg/ml), then challenged with LPS and D-galactosamine (black bars) or first pre-treated with mBPIN-GVNPs (100 μl of 1 mg/ml) via foot pad injection 1 h before treatment and then challenged with a combination of LPS and D-galactosamine (checkered bars). Data shown is from blood collected at 2 and 6 hours post-challenge. Serum cytokine levels were measured using Luminex analysis. (**a**) TNF-α levels; (**b**) IL-6 levels; (**c**) MIP-2 levels; and (**d**) KC levels. Key: ***p < 0.001, **P < 0.01, *p < 0.05, ns = not significant.

Previous studies using human BPI (hBPI) in the murine endotoxic shock model had shown a significant reduction in pro-inflammatory cytokine levels upon hBPI treatment^21^. Analysis of pro-inflammatory cytokines, including TNFα, IL-6, MIP-2 and KC showed a rise within 2 hours of LPS and D-galactosamine-treatment as compared to untreated mice (Fig. 6). mBPIN-GVNP administration prior to LPS and D-galactosamine-treatment did not affect the pro-inflammatory cytokine levels during the initial stages (first two hours) of inflammation. Interestingly the pro-inflammatory cytokine levels came down to normal within six hours in mBPIN-GVNP-treated mice compared to WT-GVNP exposed control mice. These results prove that mBPIN-GVNP can alter the serum inflammatory cytokine levels and can rescue mice from lethal endotoxic shock.

## Discussion

We have tested a novel system for producing biologically active anti-inflammatory BPI molecules by display on a buoyant protein nanoparticle in the halophilic Archaeon *Halobacterium* sp. NRC-1. *Halobacterium* lacks LPS and other inflammatory mediators which are recognized by the innate immune system, making it an ideal host for production of recombinant therapeutic proteins. *Halobacterium* GVNPs are also extremely stable and have been found to stabilize foreign antigenic proteins which have been displayed^22^,^23^. GVNPs may also be purified easily by accelerated flotation following hypotonic cell lysis which reduces the cost of purification^24^,^25^. In the present study, we have found that mouse BPI displayed on GVNPs and administered to animals by footpad injection can protect animals from LPS-induced endotoxic shock.

Previous reports have shown that recombinant human BPI may be used for the treatment of LPS induced endotoxic shock^26^,^27^. However, recombinant human BPI has a high cost of production and like most recombinant proteins will face issues of storage and stability^28^. To address these concerns, Alexander *et al.* developed adenoviral vectors expressing human BPI^21^. However, while adenoviral vectors reduce the cost of production, these vectors require time to produce the mBPI protein and may not protect some high-risk patients. The expression levels of the BPI protein also may vary from individual to individual, and the viral DNA itself may represent another potential risk.

Mouse BPI displayed on *Halobacterium* GVNPs showed antibacterial activity, providing an alternative approach for endotoxic shock treatment. This data is consistent with Lennartson *et al.* who found that the murine homolog of BPI also harbors antibacterial properties, like human BPI^29^. However, these results are not consistent with a recent report by Whittman *et al.* indicating that unlike human BPI, murine BPI does not possess antibacterial properties^30^. Murine BPI has 53% sequence identity and 71% similarity to human BPI^29^ with differences in the region utilized accounting for the observed differences.

In our study, purified mBPIN-GVNP-C3 fusion protein alone did not show significant anti-bacterial activity, but when expressed in *Halobacterium*, mBPIN-GVNPs did exhibit antibacterial properties. It is likely that the GVNP-membrane bound BPI is stabilized and more active compared to the free protein and as a result exhibits greater antibacterial properties. These findings are consistent with the location of BPI in most cells on the cell surface^31^,^32^,^33^.

In the case of endotoxic shock, patients succumb to death by multiple organ failures. Galactosamine mimics endotoxic shock by targeting hepatocytes. In our study, we have seen a marked reduction in the recruitment of neutrophils and apoptosis of liver cells following administration of BPI-GVNPs prior to exposure to LPS. These findings support previous data that LPS induced endotoxic shock in galactosamine sensitized mice is mediated through apoptosis of liver cells and that administration of mBPIN-GVNPs can inhibit the action of LPS in liver cells.

Previous reports had shown LPS neutralizing function of mBPI in cell culture model^30^. Our results show that administration of mBPIN-GVNPs before LPS treatment rescued mice from lethal endotoxic shock. Whereas treatment mBPIN-GVNPs along with or after LPS treatment didn’t rescue mice from lethal endotoxic shock. These results suggest that mBPIN-GVNPs may need to be present in the circulatory system by pretreatment for complete clearance of LPS. However, even though we see reduced inflammatory anemia as well as apoptosis following mBPIN-GVNP administration, we did not see a marked difference in serum cytokine levels during the initial stages of inflammation. Moreover, mBPIN-GVNP treatment reduced pro-inflammatory cytokine levels during the later stages of inflammation. This may be due to a time-dependent interaction of mBPIN-GVNPs with LPS. Our findings are also consistent with the possibility that LPS induced endotoxemia may not be mediated by a single burst of cytokine levels. Prolonged interaction of LPS with immune cells may be important to induce a proper endotoxic shock. mBPI-GVNPs may inhibit this response by sequestering LPS and diminishing serum pro-inflammatory cytokine levels during the later stages of inflammation.

As per our knowledge, this is the first study that validates LPS neutralizing function of mouse BPI in a mouse model of septic shock. Further studies are needed to understand the function and mechanism of action of mBPI as well as to elucidate the pathways leading to endotoxic shock. The therapeutic use of mBPIN-GVNPs produced in *Halobacterium* to combat septic shock may be promising from the standpoint of both safety and efficacy.

## Methods

### Cloning, expression and purification of GVNPs

In order to express the N-terminal 199 AA of the mouse BPI protein fragment in the *Halobacterium* host, we designed a codon-optimized mouse BPI gene fragment (Sequence ID: ref|NP_808518.1|) using an in-house Visual Basic script. The script employs a codon usage table for predicted genes in the fully sequenced *Halobacterium* sp. NRC-1 genome to replace rare codons with highly used codons^14^. The synthetic gene was generated commercially, flanked by *Afe*I cloning sites (Life Technologies, Carlsbad, CA).

**Figure 7:**
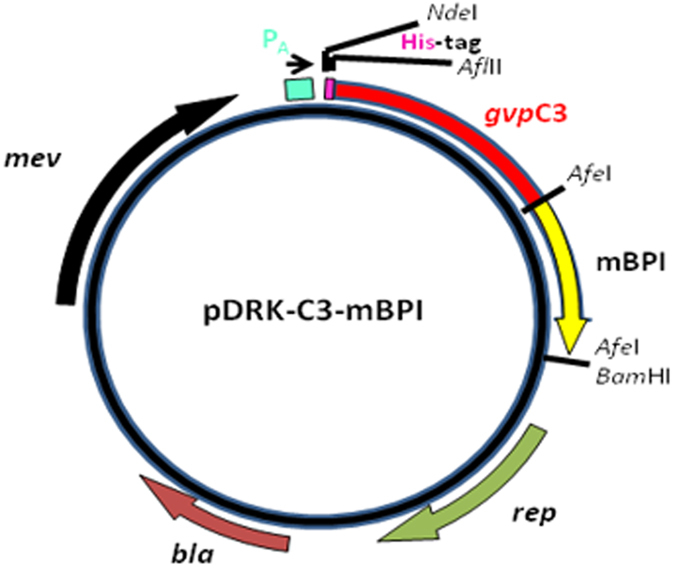
Plasmid used to transform *Halobacterium* cells to produce mBPI. Open reading frames (*gvp*C3: N-terminal *gvp*C gene region; mBPI: mBPIN gene; *rep*: origin of replication for Haloarchaea; *bla*: beta lactamase gene for ampicillin resistance and *mev*: mevinolin resistance gene) marked by arrows. Blue box: promoter; rectangular red box: 6-His tag; restriction enzymes (*NdeI*, *AfeI* and *BamH1*) cutting sites labeled with black lines.

The synthetic gene was cloned into the *Halobacterium* sp. pDRK-C3 overexpression vector, downstream of the *gvp*A promoter, and sequenced to verify correct sequence^25^. The resulting plasmid, pDRK-C3-mBPI, encoded a His-tagged fusion protein, arranged as His_6_-GvpC3-mBPIN (Fig. 7).

The pDRK-C3-mBPIN plasmid was transformed into *Halobacterium* strains for expression of the fusion protein: SD109, a strain that lacks the gas vesicle gene cluster, for the production of the free fusion protein, NRC-1, for display on GVNPs.

Expression of the mBPIN-GVP-C3 fusion protein was confirmed by Western blotting using a 1:1000 dilution of anti-His-tag primary antibody (#2635S Cell Signaling Technology, Danvers, MA) and by using a 1:2500 dilution of alkaline phosphatase-conjugated goat anti-rabbit IgG secondary antibody (Sigma-Aldrich, St. Louis, MO). Free mBPIN-GVP-C3 fusion protein was purified from *Halobacterium* sp. SD109 (pDRK-C3-mBPI) by affinity chromatography using a Ni^+2^-NTA column (GE Healthcare Life Sciences, Piscataway, NJ).

*Halobacterium* sp. NRC-1 (pDRK-C3-mBPI) was used to produce mBPIN-GVNPs and purified by floatation using the centrifugally accelerated procedure previously reported (DasSarma and Fleischman, 1995).

### Mouse model of endotoxic shock

The animal experiments were carried out in accordance with the approved guidelines of the institutional animal ethics committee at Indian Institute of Science, Bangalore, India (Registration No: 48/1999/CPCSEA). All procedures with animals were carried out in accordance with the institutional rules for animal experimentation under strict vigilance. To set up a mouse model of lethal endotoxic shock, C57BL/6J female mice aged 6 weeks were intra-peritoneally injected with 100 ng LPS from *Salmonella* Typhimurium (Sigma L6143) and 8 mg of D-galactosamine (Calbiochem). The survival of individual mice was monitored up to 5 days. Blood was collected retro-orbitally in sterile, non-heparinized 1.5 ml Eppendorf tube. Blood was processed and serum aliquots were stored at −20 °C for cytokine analysis. Mice were maintained in SPF conditions throughout the experiment.

### Bactericidal activity of mBPIN-GVNPs

Bacteria (STM 14028 and *E. coli* DH5 α) overnight cultures were subcultured at 1:100 ratio in Luria-Bertani broth for 2 hours. Log phase cells were pelleted down and washed with sterile PBS. Bacterial cell density was adjusted to 0.3 OD. 10^6^ Bacteria were incubated with PBS, 100 μg/ml of WT-GVNPs,100 μg/ml of mBPIN-GVNPs (containing approximately a total of 3 μg/ml of mBPIN-GVP-C3 fusion protein) or 6 μg/ml of mBPIN-GVP-C3 purified protein (‘free’ protein) for 2 hours at 37 °C. Serial dilutions were made in sterile PBS. Bacteria were plated on LB agar and incubated at 37 °C overnight. CFU was determined and percentage survival was calculated by normalizing CFU values with respect to CFU of PBS and WT-GVNPs for ‘free’ protein and mBPIN-GVNP fusion protein respectively.

### Anti-inflammatory activity of mBPIN-GVNPs: *in vitro*

PBMCs were isolated from healthy human volunteers using Himedia LSM as per instructors manual. 10^5^ cells were treated with 10 ng LPS or with 10 ng LPS pre-incubated with 100 μl of mBPIN-GVNPs (1 mg/ml) for 2 h. 24 h post treatment cell supernatant was collected and TNFα levels were quantified using Human TNFα ELISA development kit (MABTECH) according to the manufacturer’s protocol.

### Scanning Electron Microscopy

Bacterial samples were fixed with 2.5% (v/v) glutaraldehyde in phosphate buffered saline for 24 hours at 4 °C. Cells were dehydrated sequentially with increasing concentration of ethanol with an incubation period of 3 minutes each. Dehydrated samples were air dried and stored under vacuum until use. Samples were sputter coated using gold particles and were analyzed by Field emission SEM (FEI-SIRION, Eindhoven, Netherlands).

### Hematoxylin and eosin staining of liver sections

C57BL/6J mice were dissected 6 hours after LPS and D-galactosamine-treatment in order to evaluate the pathological effects. Tissues were fixed overnight in 4% paraformaldehyde and then dehydrated and embedded in paraffin. Paraffin-embedded liver tissues were sectioned using a microtome. Liver sections (5 μm) were processed for hematoxylin and eosin staining to identify nuclear and protein (cytoplasmic material).

### TUNEL assay to measure apoptosis in liver sections

Liver tissue sections (5 μm) were stained using propidium iodide for identifying cell viability (red) and subsequently assayed using the Dead End TUNEL assay kit (Promega Corporation)^21^ per manufactures’ specifications to analyze for nuclear DNA fragmentation (green) using a Zeiss LSM 510 confocal microscope. Representative images from ten randomly selected independent fields are shown.

### Blood collection and hematological analysis

After 6 hours of treatment with LPS and D-galactosamine, blood was collected through retro-orbital route into sterile 1.5 ml Eppendorf tube. The blood was immediately processed for analyzing blood parameters using a SysmexKX-21 Hematology Analyzer (Sysmex Corporation).

### Cytokine analysis

To determine the cytokine response, sera were tested using a Luminex analyzer (Luminex Corporation) as previously described^12^.

## Additional Information

**How to cite this article**: Balakrishnan, A. *et al.* Halobacterial nano vesicles displaying murine bactericidal permeability-increasing protein rescue mice from lethal endotoxic shock. *Sci. Rep.* **6**, 33679; doi: 10.1038/srep33679 (2016).
